# RNA-Seq Analysis of Rice Roots Reveals the Involvement of Post-Transcriptional Regulation in Response to Cadmium Stress

**DOI:** 10.3389/fpls.2015.01136

**Published:** 2015-12-21

**Authors:** Fei He, Qingquan Liu, Li Zheng, Yaqiong Cui, Zhenguo Shen, Luqing Zheng

**Affiliations:** College of Life Sciences, Nanjing Agricultural UniversityNanjing, China

**Keywords:** rice root, cadmium, RNA-seq, alternative splicing, lncRNAs

## Abstract

Widely-spread cadmium (Cd) pollution in the soil threatens both crop production and human health. How plants deal with the excess Cd are largely unknown. To evaluate the molecular mechanism by which plants respond to Cd stress, rice seedlings were treated with two concentrations of Cd and subjected to deep RNA sequencing. Comprehensive RNA-Seq analysis of rice roots under two gradients of Cd treatment revealed 1169 Cd toxicity-responsive genes. These genes were involved in the reactive oxygen species scavenging system, stress response, cell wall formation, ion transport, and signal transduction. Nine out of 93 predicted long non-coding RNAs (lncRNAs) were detected as Cd-responsive lncRNAs due to their high correlation with the Cd stress response. In addition, we analyzed alternative splicing (AS) events under different Cd concentrations. Four hundred and seventy-six differential alternatively spliced genes with 542 aberrant splicing events were identified. GO analysis indicated that these genes were highly enriched in oxidation reduction and cellular response to chemical stimulus. Real-time qRT-PCR validation analysis strengthened the reliability of our RNA-Seq results. The results suggest that post-transcriptional AS regulation may also be involved in plant responses to high Cd stress.

## Introduction

Cadmium (Cd) is one of the most toxic heavy metals to organisms. Unlike some essential heavy metals, such as copper (Cu), zinc (Zn), manganese (Mn), and nickel (Ni) that are necessary for plant growth and development, Cd is considered a non-essential metal element for plants. Widely spread Cd pollution has significantly affected human health both in terms of its direct effect on crop production and its high accumulation in the edible part of crops such as rice (Wang et al., 2015b). Previous studies have shown that excessive Cd in plants causes an accumulation of reactive oxygen species (ROS), lipid peroxidation, enzyme inactivation, and DNA and membrane damage (Hall, 2002; Boominathan and Doran, 2003), resulting in various toxicity phenotypes, such as chlorosis, wilting, growth reduction, and cell death (Sandalio et al., 2001; Rodríguez-Serrano et al., 2009). However, as signaling molecules, ROS can be produced at controlled levels and can lead to tolerance responses (Maksymiec et al., 2007; Lin and Aarts, 2012).

Plants have evolved several common mechanisms to prevent excess Cd effects, such as extracellular exudates, transport, chelation, and sequestration and repair of damaged proteins (Hall, 2002; Mendoza-Cózatl et al., 2005; Weber et al., 2006; Ahsan et al., 2009; Lin and Aarts, 2012). By inhibiting toxic metal transport, plants can reduce toxic metal influx and enhance metal removal from the cytosol (Wysocki and Tamás, 2010; Clemens et al., 2013). Several transporters involved in the acquisition, distribution and homeostasis of Cd in plants have been identified, including heavy metal ATPases (HMA), ATP-binding cassette transporters (ABC), natural resistance-associated macrophage protein (Nramp), metal transporter proteins (MTPs), and low-affinity cation transporter (LCT) (Thomine et al., 2000; Verrier et al., 2008; Yuan et al., 2012; Uraguchi et al., 2014; Slamet-Loedin et al., 2015). One recent study showed that Cd enter into rice root through OsNramp5, originally uptake transporter for essential element Mn, because of the unspecificity properties of this transporter (Sasaki et al., 2012; Yoneyama et al., 2015). The HMA family transporter OsHMA2 and OsHMA3 plays roles in translocation of Cd into shoot and sequestration of Cd into vacuole, respectively (Ueno et al., 2010; Nocito et al., 2011; Satoh-Nagasawa et al., 2012; Takahashi et al., 2012). In mature stage, OsLCT1 regulates cadmium transport into rice grains (Uraguchi et al., 2011, 2014). In *Arabidopsis*, the ABC transporter AtPDR8 is a cadmium extrusion pump conferring heavy metal resistance (Kim et al., 2007), whereas the phytochelatin transporters AtABCC1 and AtABCC2 mediate tolerance to cadmium and mercury (Park et al., 2012). Cadmium-inducible expression of the ABC-type transporter AtABCC3 increases phytochelatin-mediated cadmium tolerance in *Arabidopsis* (Brunetti et al., 2015). Glutathione (GSH) and phytochelatins (PCs) chelating represent another detoxification strategy in plant cells (Cobbett and Goldsbrough, 2002).

Microarray data provides highly useful information on the plant responses to Cd stress at the transcriptomic level. Genes related to Cd response have been identified by various microarray analyses in plants, including transcriptomic analysis of the response to Cd stress in *Arabidopsis* roots, comparative transcriptomic analysis of Cd-treated roots of *A. thaliana* and the Cd-hypertolerant metallophyte *Arabidopsis halleri*, a time course analysis of gene regulation under Cd stress in rice shoots and roots, a microarray-based analysis of cadmium-responsive microRNAs in rice, and an analysis of early transcriptomic responses to Cd in rice roots (Herbette et al., 2006; Weber et al., 2006; Ogawa et al., 2009; Zhao et al., 2009; Ding et al., 2013; Lin et al., 2013). These transcriptomic analyses using microarray have given rise to a view of Cd-responsive gene expression in plants. However, compared with the high-throughput sequencing strategies, the microarray based transcriptomic strategy may lead to the loss of some important candidate genes during screening (Weber et al., 2006). At present, still a few transcriptomic studies focusing on genes involved in the plant response to Cd have been conducted using high-throughput sequencing approaches (Tang et al., 2014; Peng et al., 2015; Xu et al., 2015). While some candidate transcription factors (TFs) involved in Cd tolerance have been identified using chip strategy (DalCorso et al., 2010), the regulatory mechanisms involved are still largely unknown. Expression of several genes change significantly under Cd stress, and the identification of TFs including MYB, bZIP, ethylene-responsive factor (ERF), and WRKY by transcriptomic studies in several plants, including *Arabidopsis*, rice and barley, suggest that transcriptional regulation plays a pivotal role in the plant response to heavy metal stress (DalCorso et al., 2010). However, the alternative splicing regulatory role as a crucial post-transcriptional mechanism in response to Cd stress is less understood.

The expression of genes is highly regulated at both transcriptional and post-transcriptional level. Post-transcriptional regulations of gene expression occur at the levels of pre-messenger RNA (mRNA) processing (capping, splicing, and polyadenylation), mRNA stability, and mRNA translation (Floris et al., 2009). Alternative splicing (AS) is a typical post-transcriptional regulation. Recent studies indicate that AS play an important role in plant in response to abiotic stresses. The regulatory role of pre-mRNA splicing in temperature (Chang et al., 2014; Capovilla et al., 2015; Filichkin et al., 2015a), nutrient deficiency (Li et al., 2013) and salt (Ding et al., 2014; Feng et al., 2015) stresses has been reported.

High-throughput sequencing strategies have provided a new perspective on unknown transcript dynamics under specific stresses. In this study, we used RNA-Seq to understand the mechanisms of Cd toxicity, cellular detoxification and protection pathways in response to Cd in rice roots. Here, we found candidate genes associated with the reactive oxygen species (ROS) scavenging system, stress related proteins, transporters, TFs, signaling transduction, cell wall metabolism, and long non-coding RNAs (lncRNAs).

The results of our study provide a new overview of transcriptomic responses to Cd stress. These responsive genes may contribute to a reduction in Cd toxicity and rice tolerance to Cd stress. Furthermore, we examined the expression patterns of lncRNAs and revealed the genome-wide dynamic changes in alternative splicing in response to Cd stress. The transcriptional and post-transcriptional alterations in rice roots under Cd stress provide a more comprehensive understanding of the plant responses to Cd stress.

## Materials and methods

### Plant materials, growth conditions, and treatments

Rice (*Oryza sativa* spp. japonica cv. Nipponbare) seeds were sown on mesh floating in a 0.5 mM calcium chloride (CaCl_2_) solution and maintained for 2 days at 25–30°C in the dark, thereby inducing germination. Seedlings were transferred into Kimura B nutrient solution containing the macronutrients (mM): (NH_4_)_2_SO_4_ (0.18), MgSO_4_·7H_2_O (0.27), KNO_3_ (0.09), Ca(NO_3_)_2_·4H_2_O (0.18), and KH_2_PO_4_ (0.09); and the micronutrients (μM): MnCl_2_·4H_2_O (0.5), H_3_BO_3_ (3), (NH_4_)_6_Mo_7_O_24_·4H_2_O (1), ZnSO_4_·7H_2_O (0.4), Fe-EDTA (20), and 0.2 μM CuSO_4_·5H_2_O (Zheng et al., 2012). The pH of the nutrient solution was adjusted to 5.5, and held under normal greenhouse conditions with illumination provided by cool-white fluorescent lamps. Growth conditions were as follows: 27/24°C day/night temperatures, 60–80% relative humidity, and a 14/10-h day/night photoperiod. Fifteen-day-old rice seedlings were treated with or without 10 and 100 μM solutions of Cd (II) hydrochloride (CdCl_2_) for 24 h. Following Cd treatment, roots were harvested for RNA extraction and subsequent analysis. Samples were stored at −80°C if not immediately used for RNA isolation. All experiments were performed at least twice with three biological replicates each, and representative results of one experiment are shown.

### RNA isolation, RNA-Seq library preparation and sequencing

Total RNA for RNA-Seq was extracted from roots using a plant RNA kit (Omega, USA). Purified RNA was analyzed either using a ND-8000 spectrophotometer (Nanodrop Technologies, Inc., Wilmington, DE, USA), by agarose gel electrophoresis, or using a 2100-Bioanalyzer (Agilent Technologies, Santa Clara, CA, USA) to determine the RNA quantity. Those RNA samples with no smear seen on agarose gels, a 260/280 ratio above 2.0, and a RNA integrity number greater than 8.0 were used.

For RNA-Seq analysis, we mixed three replication samples for each treatment into one, and total RNA samples were then sent to Genergy Biotechnology Corporation (http://www.genenergy.cn/) for sequencing. The TruSeq RNA sample preparation kit was used for mRNA purification, fragmentation, and first- and second-strand cDNA synthesis. Double-stranded cDNAs were then purified for end repair, dA tailing, adaptor ligation, and DNA fragment enrichment. The libraries were sequenced as 101-bp paired-end reads using Illumina Hiseq2500 according to the manufacturer's instructions. Illumina reads of all samples had been submitted to the Sequence Read Archive at the National Center for Biotechnology Information (http://www.ncbi.nlm.nih.gov/sra) under accession number SRP058434.

### Validation of gene expression

To validate the RNA-Seq results, the expression of selected up- or down-regulated genes, long non-coding RNA genes and alternative splicing events were confirmed by quantitative reverse transcription–polymerase chain reaction (qRT-PCR) or semi-quantitative RT-PCR analysis. After 15-day-old rice seedlings were treated with or without 0.1, 1, 10, and 100 μM CdCl_2_ solutions of for 24 h, the roots samples were harvested for RNA extraction. After RNA extraction, genomic DNA was removed with the Rnase Free Dnase Set (Omega, USA) following the manufacturer's instructions. RNA was reverse-transcribed using a Super Reverse transcription kit (BioTeke, China). The quantitative real-time PCR was performed on a 7500 PCR system (Applied Biosystems, USA) to confirm the RNA-seq results with the primer sets shown in Supplemental Tables S1–S3. The PCR protocol was as follows: initial denaturation at 95°C for 30 s, followed by 95°C for 5 s, and 60°C for 34 s in a 40-cycle reaction, then followed by dissociation stage. *ACTIN* was used as an internal standard (Liu et al., 2015). 2 ^−ΔΔCt^ method calculation was used for data analysis. Moreover, we also performed qRT-PCR validations on two genes showing no expression changes upon Cd stress (Supplemental Table S31).

### Mapping reads to the *Oryza sativa* genome with combined gene annotation

Complete known gene/transcript annotation information for *Oryza sativa* was integrated from RAPDB (http://rapdb.dna.affrc.go.jp/download/archive/irgsp1/IRGSP-1.0_representative_2015-03-31.tar.gz,IRGSP-1.0_predicted_2015-03-31.tar.gz) and MSU 7.0 (ftp://ftp.plantbiology.msu.edu/pub/data/Eukaryotic_Projects/o_sativa/annotation_dbs/pseudomolecules/version_7.0/all.dir/all.gff3) databases using Cuffcompare method in Cufflinks (Trapnell et al., 2010; Kawahara et al., 2013; Sakai et al., 2013). For each sample, raw paired-end reads were quality- and adaptor-trimmed using Trim Galore (-q 25, -length 30). Next, clean reads were aligned to the Os-Nipponbare-Reference-IRGSP-1.0 reference genome (http://rapdb.dna.affrc.go.jp/download/archive/irgsp1/IRGSP-1.0_genome.fasta.gz) using TopHat2 with the “-G” option of the above merged reference gene GTF file (Trapnell et al., 2009).

### Long non-coding RNA prediction

“Cufflinks-Cuffmerge-Cuffcompare” pipeline was used to identify novel transcripts. For each sample, mapped reads were assembled using the Reference Annotation Based Transcript assembly strategy from Cufflinks utilizing the “-g” option of the above merged reference gene GTF file. Three sets of separately assembled transcripts were merged together into the final set of assembled transcripts using Cuffmerge. All transcripts were then compared to all known gene annotations of RAPDB and MSU using Cuffcompare. Unknown intergenic transcripts, intron transcripts, antisense exon transcripts overlapping within reference exons and antisense intron transcripts overlapping within reference introns were characterized as preliminary candidate lncRNA transcripts. Novel transcripts, which were single-exon or short, could be false positives due to sequencing or alignment errors. In order to effectively distinguish lncRNAs from protein-coding transcripts, two software tools named CPC and CNCI were implemented to predict plant lncRNAs. CPC (Coding Potential Calculator: http://cpc.cbi.pku.edu.cn/) was a support vector machine-based classifier which assessed the protein-coding potential of a transcript based on quality, completeness, and sequence similarity of its open reading frame to known proteins in NCBI NR database (Kong et al., 2007). CNCI (Coding-Non-Coding Index: http://www.bioinfo.org/software/cnci) was also a support vector machine-based classifier which effectively distinguished protein-coding and non-coding transcripts based on the usage frequency of adjoining nucleotide triplets between coding and non-coding region sequences (Sun et al., 2013). For either coding/non-coding classifier, the smaller than zero its predicted score was, the more reliable prediction of lncRNA transcript was. In order to reduce false positives, candidate lncRNAs were predicted by satisfying the following criteria: Exon number ≥ 2, RNA length ≥ 200 nt, CPC score < 0, and CNCI score < 0.

### Differential expression analysis

Raw read count of each known or predicted gene was generated using HTSeq with union-count mode (Anders et al., 2014). Within the merged reference gene annotation, a small number of genes overlap, either on the same strand or on different strands. To avoid erroneous counting of sense and antisense reads, ambiguous aligned reads from overlapped regions were excluded. After normalization by estimating the dispersion factors in DESeq R package, normalized read count table was used for determining statistical significance (Anders and Huber, 2010). Differentially expressed genes exhibiting 2-fold changes and Benjamini and Hochberg-adjusted *P*-values (FDR) ≤ 0.05 were selected. If the gene normalized read count value was close to 0, log2 (normalized read count+1) was used to compute the fold change. In order to show expression increased or decreased trend of these differential expressed genes with the rise of Cd concentration, the log2-transformed normalized count value of each gene was standardized to the same scale with a mean of 0 and standard deviation of 1, and then a heatmap was generated using pheatmap R package. For Cd up- or down-regulated gene set enrichment analysis, agriGO was used to detect the significantly enriched GO terms compared with the genome-wide background. KEGG pathway enrichment analysis was performed using the Hypergeometric test in R. Significantly enriched GO terms and KEGG pathways were also selected by a threshold FDR (adjusted *P*-value) ≤ 0.05.

### Differential alternative splicing analysis

Alternative splicing events (AS) are classified into five broad categories including skipped exon, retained intron, alternative 5′ splice site, alternative 3′ splice site and mutually exclusive exon (Barbazuk et al., 2008). For each sample, known and novel AS event was extracted from aligned BAM file and the above merged reference gtf file using rMATS (Shen et al., 2014). rMATS also quantified AS event of which exon or intron was included by inclusion junction counts (IJC) and of which exon or intron was skipped by skipping junction counts (SJC). Expressed alternative splicing events were declared if IJC ≥ 1 and SJC ≥ 1. To identify differential alternative splicing (AS) events between control and Cd-treated conditions, rMATS evaluated whether the difference in the exon or intron inclusion level of AS event between two conditions exceeds a stringent threshold (|IncLevelDifference|≥ 0.1 and *P* ≤ 0.05).

## Results

### Alignment and assembly of RNA-Seq datasets

**Figure 1:**
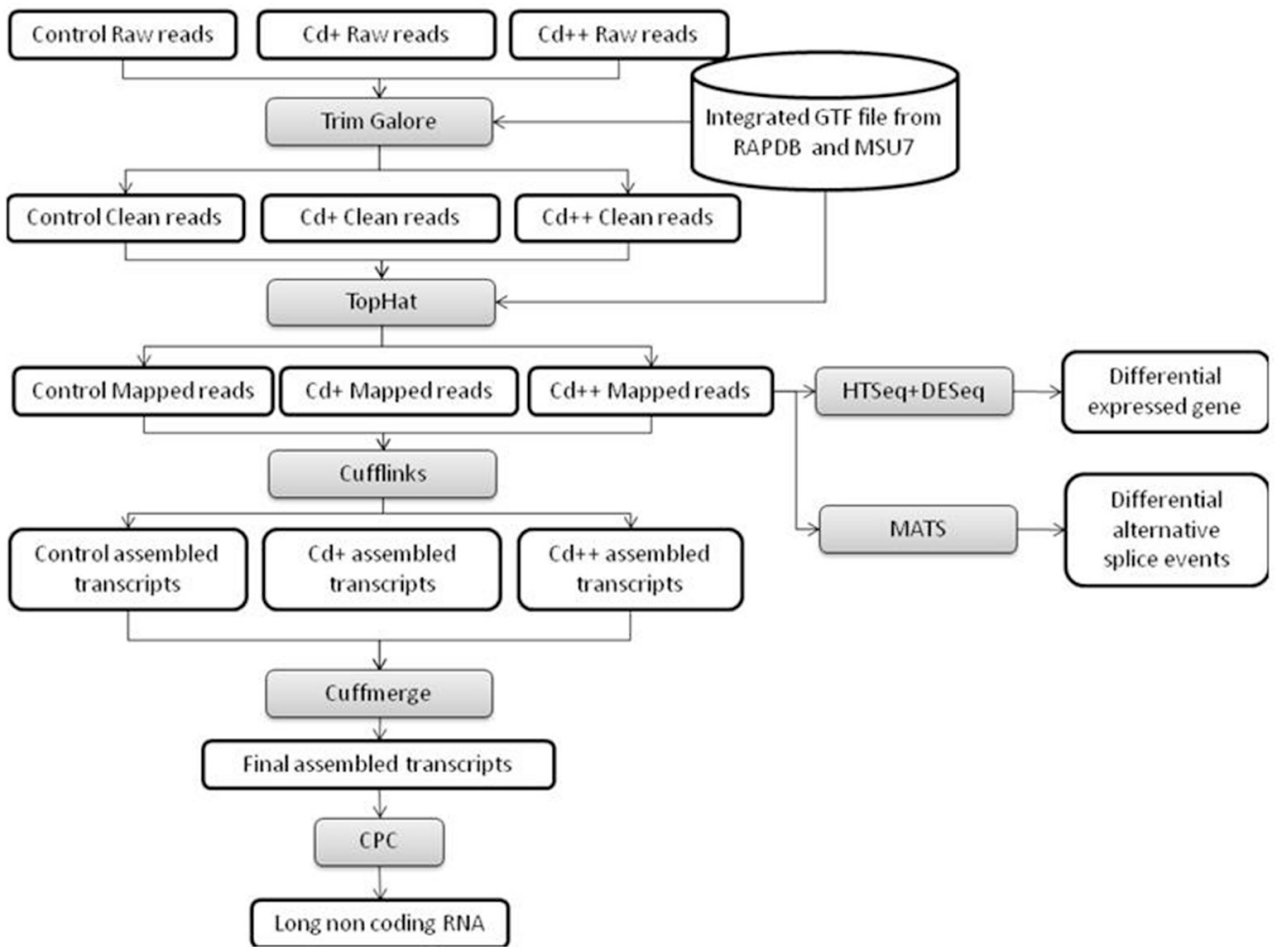
**Flowchart of our RNA-seq data analysis workflow**. High-level representation of the pipeline workflow for processing RNA-Seq data.

To gain additional insight into the rice transcriptomic response to environmental Cd stress, 15-day-old rice seedlings were treated with 10 or 100 μM solutions of Cd^2+^, or without Cd (control), for 24 h, at which point root samples were harvested and labeled as Cd+, Cd++, and control, respectively. These samples were used for 101 bp paired-end (PE) deep sequencing on an Illumina HiSeq 2500 platform. After the adaptor and low-quality sequences of pair-end reads were trimmed, in total, ~218 million clean reads (~21.5 Gb) were obtained, with an average of ~72.8 million reads (~6.9 Gb) per sample. Our workflow for analysis of RNA-Seq data is illustrated in Figure 1. An average of ~68.9 million clean reads per sample, corresponding to ~94.6% of the total clean reads per sample, were aligned to the Os-Nipponbare-Reference-IRGSP-1.0 reference genome using TopHat2. Among the mapped reads per sample, ~96.7% were uniquely aligned, and the remaining 3.3% were multiple mapped reads (Table 1). For each sample, the resulting aligned reads were then analyzed with Cufflinks, which assembles the aligned reads into transcripts. The assembled transcripts were further filtered based on greater than 0 FPKM (Fragments Per Kilobase Million) expression level. The remaining transcripts were processed separately by comparing our merged reference transcript annotation using Cuffcompare and further classified into four main categories: known reference transcript, novel isoform transcripts, antisense transcripts, and intergenic transcripts. An average 32.7% of 113,001 merged known reference transcripts per sample was expressed. Averagely, 8908 novel isoform transcripts, 157 antisense transcripts, and 754 intergenic transcripts for each sample were also detected (Table 2).

**Table 1:** **Statistics of the Read Alignments in the RNA-Seq study**.

**Library**	**Control**	**Cd+**	**Cd++**
Raw reads (R) (Gb)	76,676,410 (7.7 Gb)	85,050,612 (8.6 Gb)	76,051,916 (7.7 Gb)
Clean reads (C) (Gb)	70,177,244 (6.7 Gb)	77,914,054 (7.4 Gb)	70,021,098 (6.7 Gb)
Total mapped reads (T) (T/C%)	66,759,177 (95.1%)	73,903,926 (94.9%)	65,970,820 (94.2%)
Unique mapped reads (U) (U/C%)	64,533,632 (92.0%)	71,508,179 (91.8%)	63,670,122 (90.9%)
Multiple mapped reads (M) (M/C%)	2,225,545 (3.1%)	2,395,747 (3.1%)	2,300,698 (3.3%)

**Table 2:** **Number of transcripts detected by annotation classification reported by Cufflinks and cuffcompare**.

**Cufflinks transcripts**	**Control**	**Cd+**	**Cd++**
Total reference transcripts (T)	113,001		
Known reference transcripts (K) (K/T%)	36,923 (32.7%)	37,028 (32.8%)	36,743 (32.5%)
Novel isoform transcripts (N)	8906	8868	8950
Antisense transcripts (A)	166	175	157
Intergenic transcripts (I)	721	732	810

### Identification of differentially expressed genes

**Figure 2:**
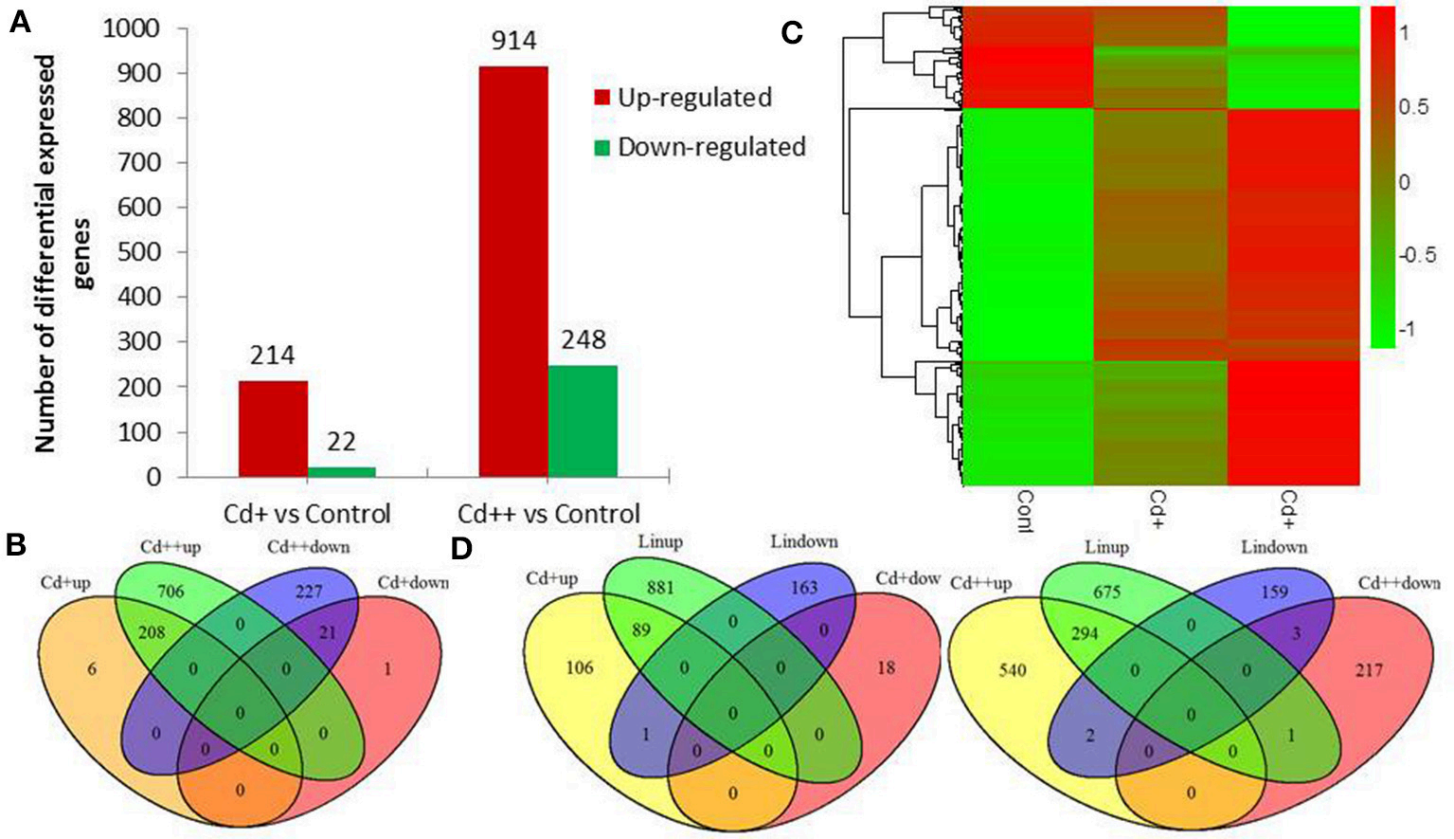
**Differentially expressed genes of *Oryza sativa* in response to Cd stress from RNA-seq data. (A)** Summary of significant up- and down-regulated genes between two Cd concentration gradient treated roots. **(B)** Venn diagram analysis showed a total of 1169 differentially expressed genes in different conditions. **(C)** Heatmap showed expression increased or decreased trend of total differential expressed genes with the rise of Cd concentration after scaling each gene to a mean of 0 and standard deviation of 1. **(D)** Overlap of differential expressed RAPDB genes from RNA-Seq and Lin et al microarray study. Total number of RAPDB genes was 45,990. The *P*-value of overlap significant statistical test was 2.2E-16 and 2.2E-16 by Fisher exact test, respectively.

**Figure 3:**
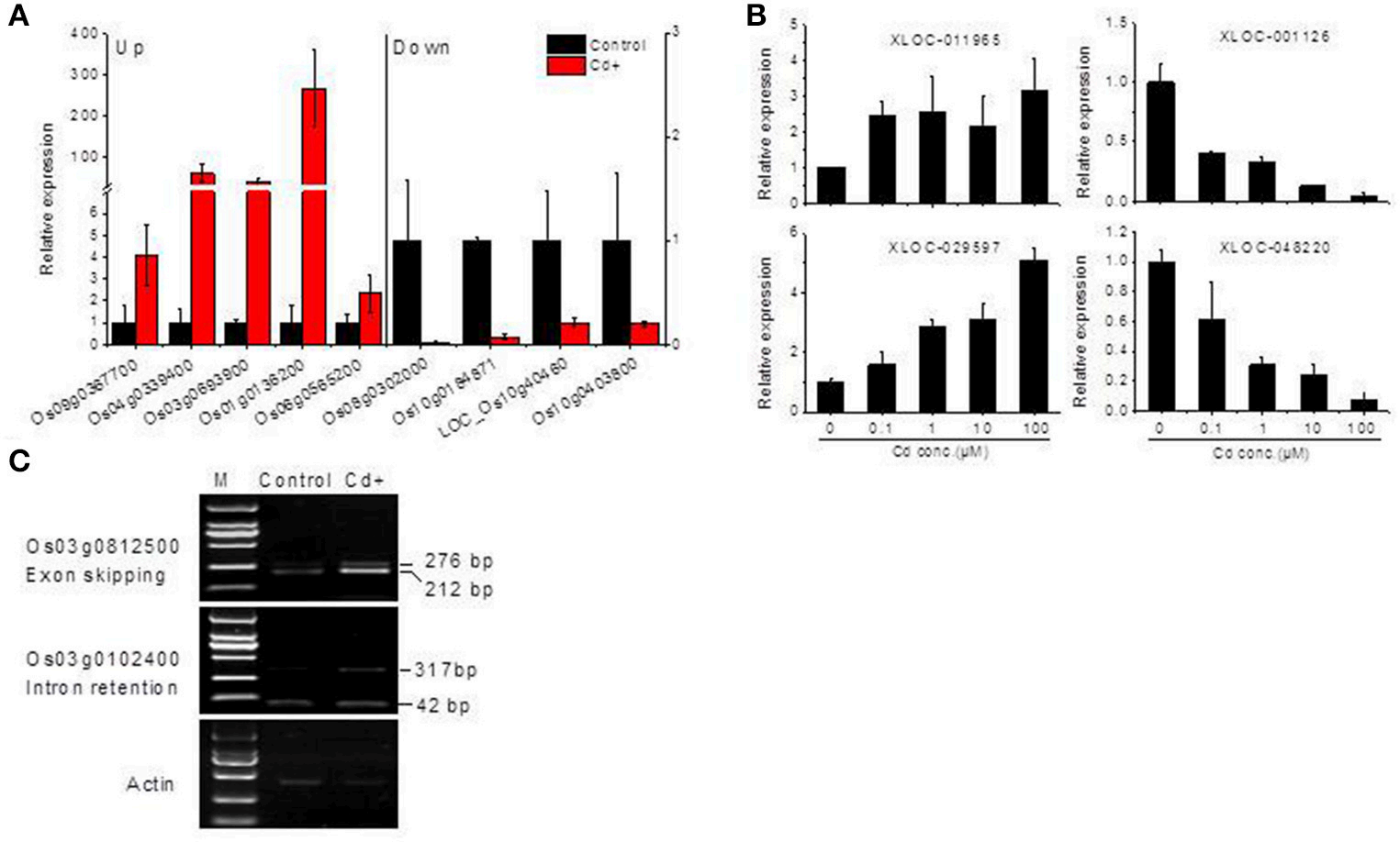
**Validation of RNA-Seq data by real-time qRT-PCR or RT-PCR. (A)** Validation of several differentially expressed Cd up- or down-regulated genes by qRT-PCR. **(B)** qRT-PCR detection of long non-coding RNAs expression under Cd stress. **(C)** Semi-quantitative PCR detection of alternative splicing event analysis under Cd stress. Rice seedling roots were collected 24 h after 10 μM Cd (Cd+) exposure in **(A,C)**, or treated with 0.1, 1, 10, and 100 μM Cd for **(B)**, see details in the Materials and Methods.

Differential expression genes (DEGs) were identified between Cd+ or Cd++ and control samples using DESeq. A total of 1169 DEGs of *Oryza sativa* in response to Cd stress from the RNA-Seq data were identified. Under Cd+ treatment, 214 were up-regulated while 22 were down-regulated (Figure 2A, Supplemental Tables S4, S5). Under Cd++ treatment, 914 genes were up-regulated, and 248 were down-regulated (Figure 2A, Supplemental Tables S6, S7). These results suggest that most up- and down-regulated genes are common under the two gradients of Cd treatment. 208 out of 214 regulated genes and 21 out of 22 repressed genes regulated by Cd+ treatment were also among the up- and down-regulated genes regulated by Cd++ treatment (Figure 2B). In addition to the significantly increased number of up- and down-regulated genes after Cd++ compared with Cd+ treatment, the expression ratio also changed significantly between the two concentrations of Cd relative to the control, indicated by the Heatmap analysis (Figure 2C). We compared these DEGs with results from a previous study (Lin et al., 2013) that used an Agilent two-color Rice Oligo DNA Microarray 44 K designed for RAP-DB genes. 89 (45.4%) out of 196 Cd+ up-regulated genes and 294 (35.2%) out of Cd++ 836 up-regulated genes identified in our RNA-Seq assay were also found in the microarray datasets, while in contrast, most of the down-regulated genes varied between the two assays (Figure 2D). In addition to the differences in culture conditions and the rice cultivars we used, the differences between two-color microarray and RNA-Seq technologies may also lead to the discrepancy on some DEGs results. Real-time qRT-PCR analysis was also performed to validate some up- and down-regulated genes expression data according to the RNA-Seq results (Figure 3A).

**Figure 4:**
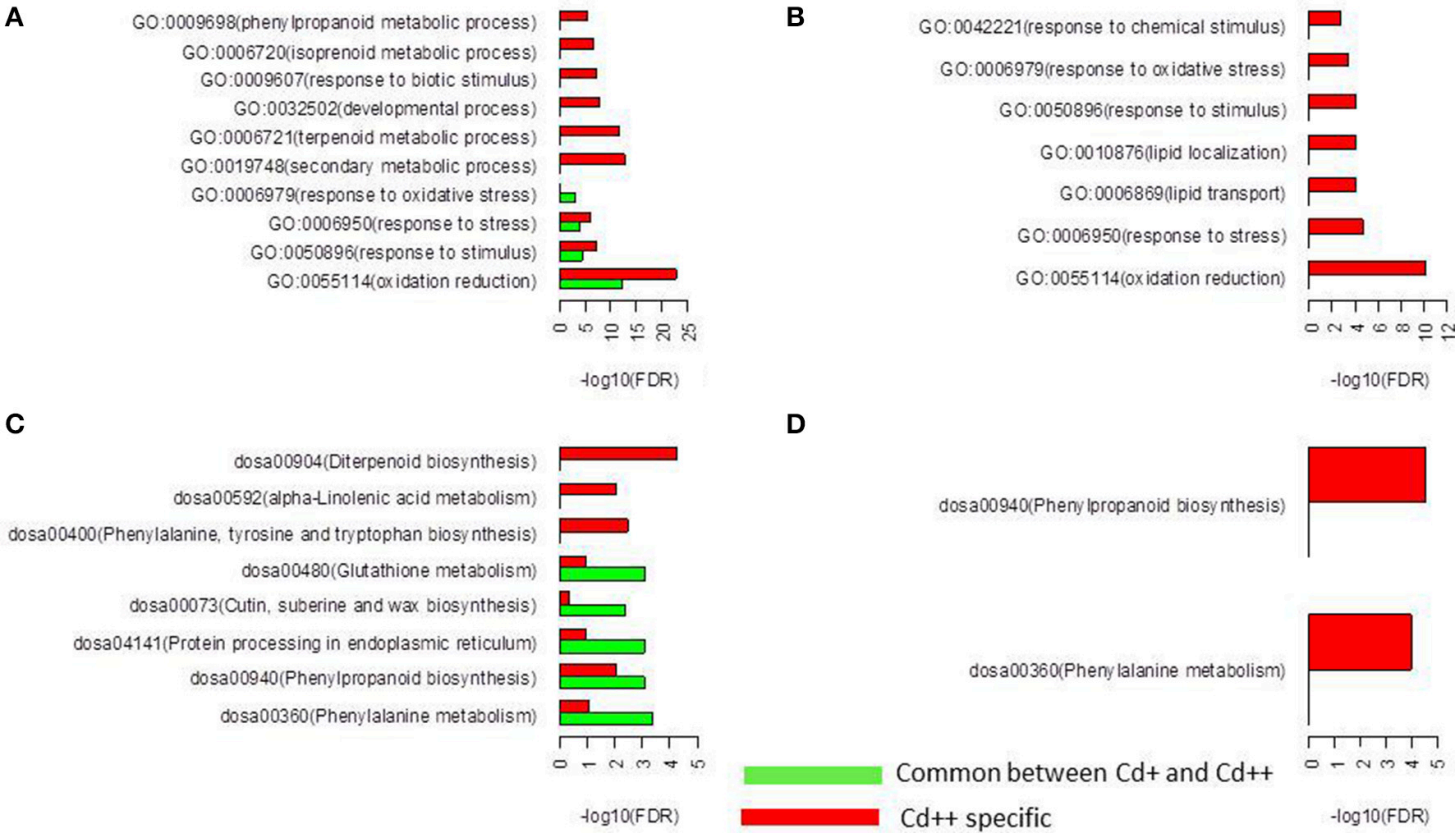
**Function enrichment analysis of differentially expressed genes. (A)** Gene Ontology (GO) enrichment analysis for the up-regulated genes. Only the top false discovery rate (FDR) ranked 10 enrichment of GO terms from “biological process” category were listed. **(B)** GO enrichment analysis for the down-regulated genes. Only “biological process” category were listed. **(C)** KEGG pathway analysis for the up-regulated genes. **(D)** KEGG pathway analysis for the down-regulated genes.

To identify possible biological processes or pathways that were altered in Cd stress treatment and between the two levels of Cd stress, Gene Ontology (GO) and KEGG pathway functional enrichment was performed using an FDR adjusted *p* ≤ 0.05 as the cutoff. Our analysis revealed that 208 commonly up-regulated DEGs were highly enriched in oxidation reduction, response to stimulus, response to stress, response to oxidative stress, phenylalanine metabolism, phenylpropanoid biosynthesis, protein processing in endoplasmic reticulum, cutin, suberine and wax biosynthesis and glutathione metabolism. In contrast, there were no significantly enriched biological processes and pathways in 21 commonly down-regulated DEGs. Moreover, 706 Cd++ specific up-regulated DEGs were highly enriched in oxidation reduction, response to biotic stimulus, response to stimulus, response to stress, cell wall organization or biogenesis, oxidation reduction, secondary metabolic process, lipid metabolism, regulation of transcription, phenylalanine, tyrosine and tryptophan biosynthesis, phenylpropanoid biosynthesis, alpha-Linolenic acid metabolism, and diterpenoid biosynthesis. Two hundred and twenty-seven Cd++ specific down-regulated DEGs were highly enriched in oxidation reduction, response to stress, lipid transport, lipid localization, response to stimulus, response to oxidative stress, response to chemical stimulus, phenylalanine metabolism and phenylpropanoid biosynthesis. It was noteworthy that the common significantly enriched GO terms and KEGG pathways identified by separate enrichment analysis strategy for up- and down-regulated genes were oxidation reduction (37 up- and 11 down-regulated genes), response to stimulus (66 up- and 19 down-regulated genes), response to stress (55 up- and 19 down-regulated genes), response to oxidative stress (12 up- and 8 down-regulated genes), phenylalanine metabolism (17 up- and 5 down-regulated genes), and phenylpropanoid biosynthesis (21 up- and 7 down-regulated genes). Within these functional GO terms and KEGG pathways, more genes were found to be induced than repressed, particularly, 8 up- and 5 down-regulated peroxidase genes were in common. Globally, the above results indicated that the gradual increase of Cd concentration had significant effects on cell wall biogenesis, lipid metabolism and regulation of transcription (Figure 4, Supplemental Tables S8–S11).

### Cd stress regulated transcription factors

**Figure 5:**
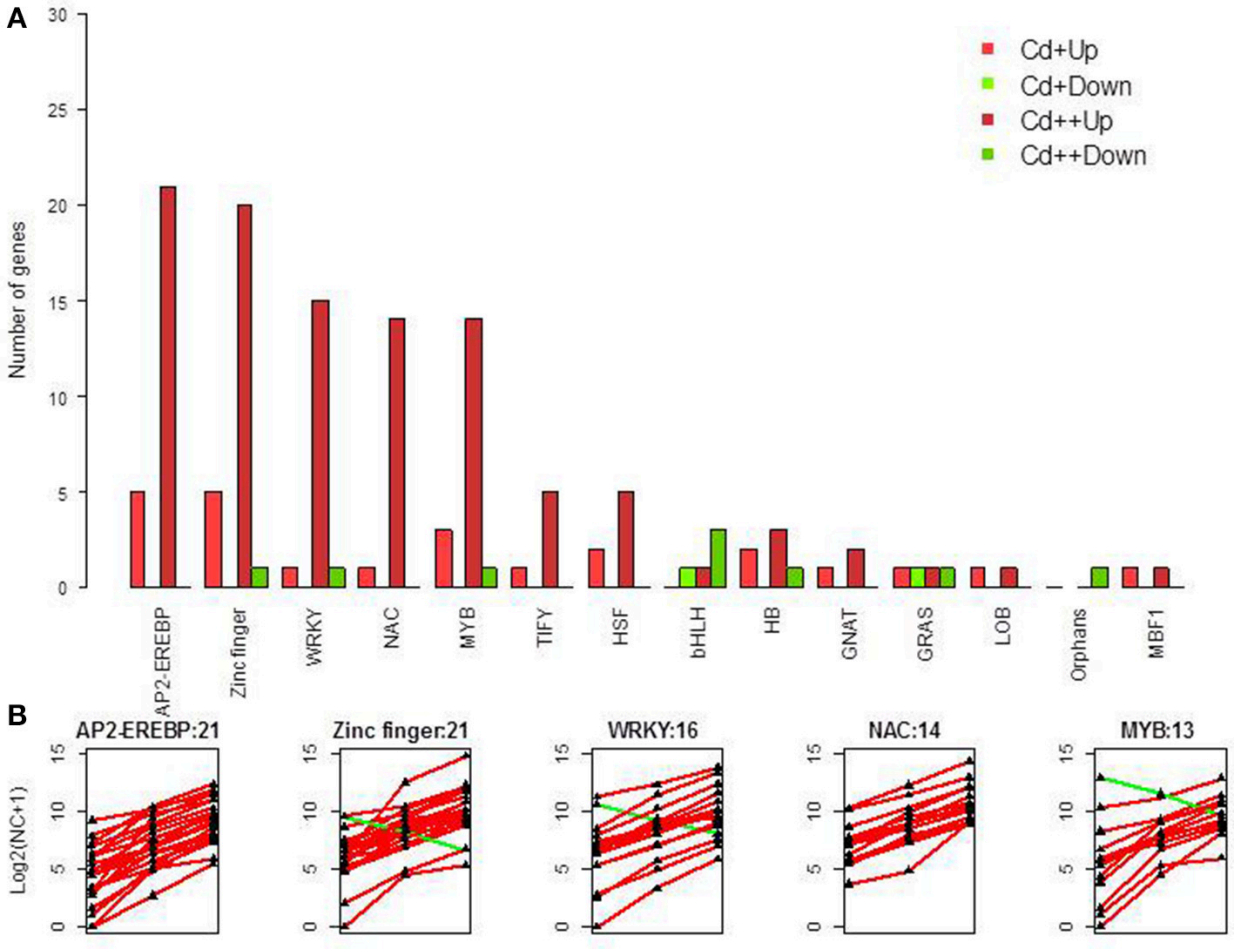
**Differentially expressed transcription factors. (A)** Summary of significant up- and down-regulated transcription factors under two different levels of Cd treatment compared with control in rice roots. **(B)** Gene expression pattern of five transcription factor classes, which contained most DGEs.

Given that TFs appear to have a major effect on the network of Cd-responsive genes, one objective of our work was to identify Cd regulated TFs. In total, 110 differentially expressed TF genes were identified following Cd treatment (Figure 5A, Supplemental Table S12). 20 up-regulated genes and 1 down-regulated gene were characterized as zinc finger TFs. 21 up-regulated genes were characterized as AP2-EREBP TFs. 15 up-regulated genes and 1 down-regulated gene were characterized as WRKY TFs. 14 up-regulated genes were characterized as NAC TFs. 12 up-regulated genes and 1 down-regulated gene were characterized as MYB TFs (Figure 5B). Moreover, a relatively small group of TIFY, HSF, bHLH, HB, GRAS, GNAT, LOB, MBF1, and Orphans TF family members were also present in the Cd-regulated TF family (Figure 5).

### Identification of long non-coding RNAs

**Figure 6:**
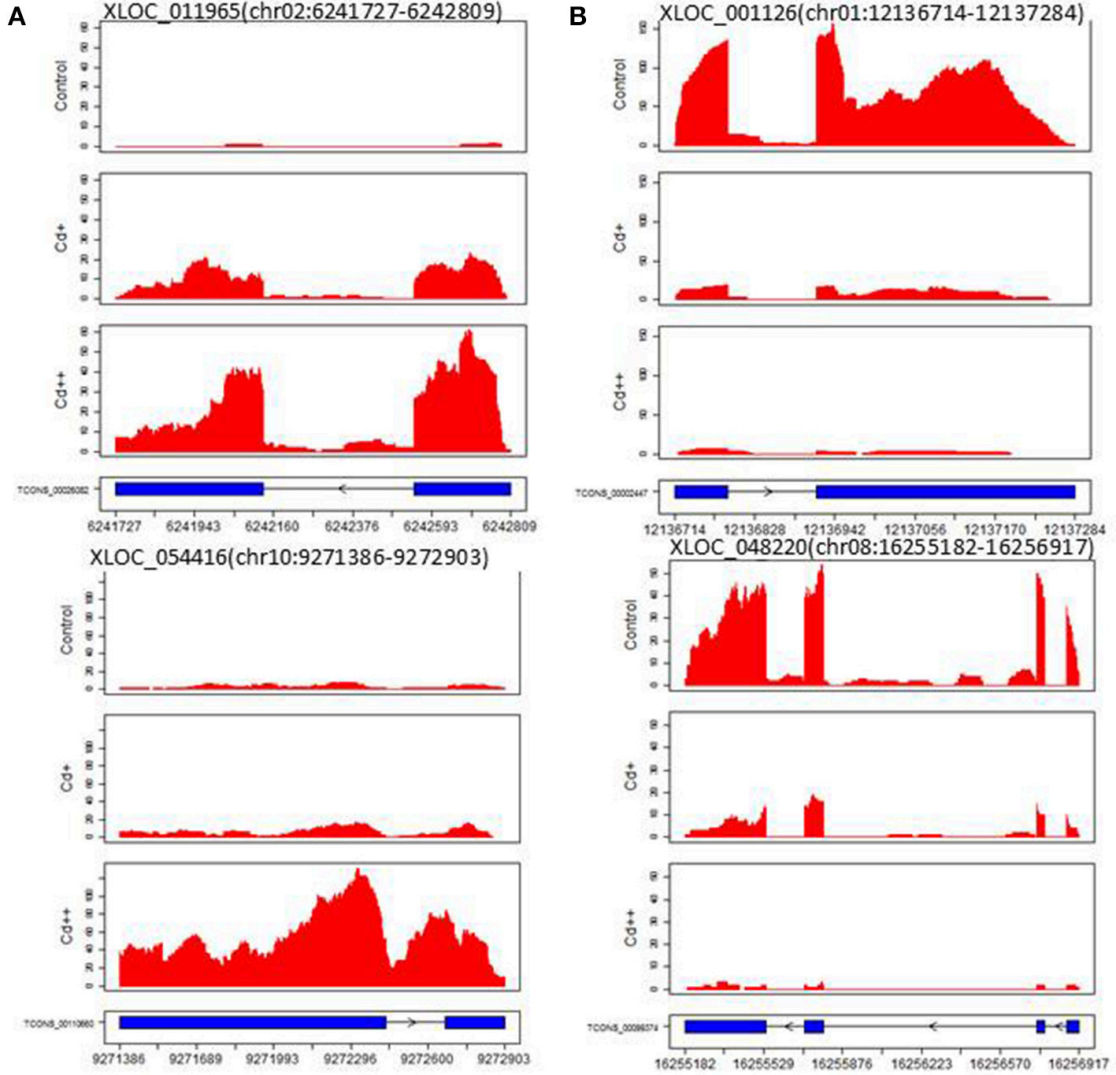
**Read coverage signal maps and exon-intron structure of long non-coding RNAs. (A)** Two up-regulated lncRNAs: XLOC_011965 and XLOC_054416. **(B)** Two down-regulated lncRNAs: XLOC_001126 and XLOC_048220.

A computational prediction pipeline of lncRNAs based on RNA-Seq data was performed (see Materials and Methods). In total, 93 novel genes corresponding to 122 transcripts were predicted as lncRNAs (Supplemental Table S13). According to their genomic location, 29 antisense lncRNAs were transcribed from the antisense strand of protein-coding genes, of which 13 were close to the 5′-untranslated region (UTR), 14 overlapped with middle coding exon region, only 2 were close to the 3′ UTR. 8 bidirectional lncRNAs were transcribed head-to-head with a protein-coding gene within 1 kb. The remaining 56 intergenic lncRNAs were located at least 1 kb away from a protein-coding gene. A total of 9 differentially expressed genes were detected between Cd-treated and control samples (Table 3). Among them, 5 were commonly induced and 4 were commonly repressed by Cd. Typically, read coverage signal maps of 2 up-regulated and 2 down-regulated lncRNAs were shown (Figures 6A,B). Real-time qRT-PCR validation performed in four of them confirmed their Cd-regulated expression pattern (Figure 3B).

**Table 3:** **Differentially expressed lncRNAs between Cd-treated and control samples**.

**LncRNA gene and transcript id**	**Position and strand**	**Classification**	**Log2-fold-change**	
			**Cd+**	**Cd++**
XLOC_001135//TCONS_00002448	chr01:12150245–12151434(+)	Intergenic	−1.72↓^*^	−3.202↓^**^
XLOC_026102//TCONS_00055364	chr04:16429867–16432135(−)	Antisense (close to 5′UTR)	2.083↑^*^	4.86↑^**^
XLOC_026102//TCONS_00055365	chr04:16429867–16432135(−)	Antisense (close to 5′UTR)	2.083↑^*^	4.86↑^**^
XLOC_039204//TCONS_00081563	chr07:358916–359977(+)	Antisense (close to 5′UTR)	1.497↑	5.385↑^**^
XLOC_048220//TCONS_00099374	chr08:16255182–16256917(−)	Intergenic	−2.143↓^*^	−4.354↓^**^
XLOC_054416//TCONS_00110660	chr10:9271386–9272903(−)	Intergenic	0.941↑	4.35↑^**^
XLOC_062733//TCONS_00126507	chr11: 28697042–28697508(−)	Intergenic	−2.103↓^**^	−6.443↓^**^
XLOC_001126//TCONS_00002447	chr01:12136714–12137284(+)	Intergenic	−3.096↓^**^	−4.657↓^**^
XLOC_011965//TCONS_00026082	chr02:6241727–6242809(−)	Intergenic	5.275↑^**^	6.727↑^**^
XLOC_029597//TCONS_00062817	chr05:20601026–20601447(−)	Antisense (mainly overlap to coding region)	2.216↑	5.693↑^**^

*↑|log2-fold-change|≥1, upregulated*.

*↓|log2-fold-change| ≤ -1, downregulated*.

* *P-value or FDR ≤ 0.05*.

** *P-value or FDR ≤ 0.01*.

### Cd regulates alternative splicing

**Figure 7:**
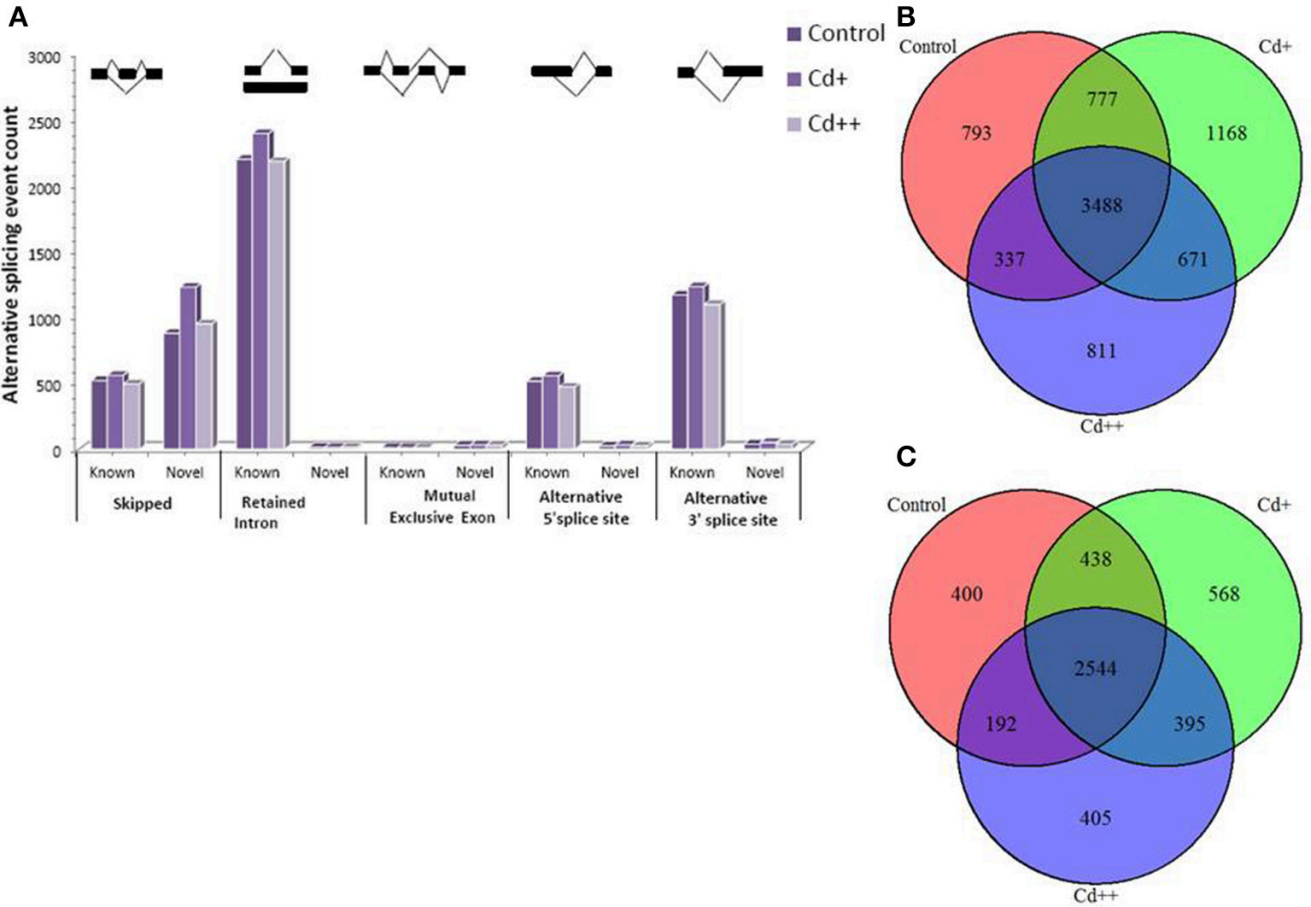
**Expressed alternative splicing events from RNA-seq data and comparsion analysis between Cd-treated and control. (A)** Statistics of detected known and novel expressed alternative splicing events in control, Cd+, Cd++. **(B)** Overlap of expressed AS events between Cd-treated and control. **(C)** Overlap of AS genes between Cd-treated and control.

**Figure 8:**
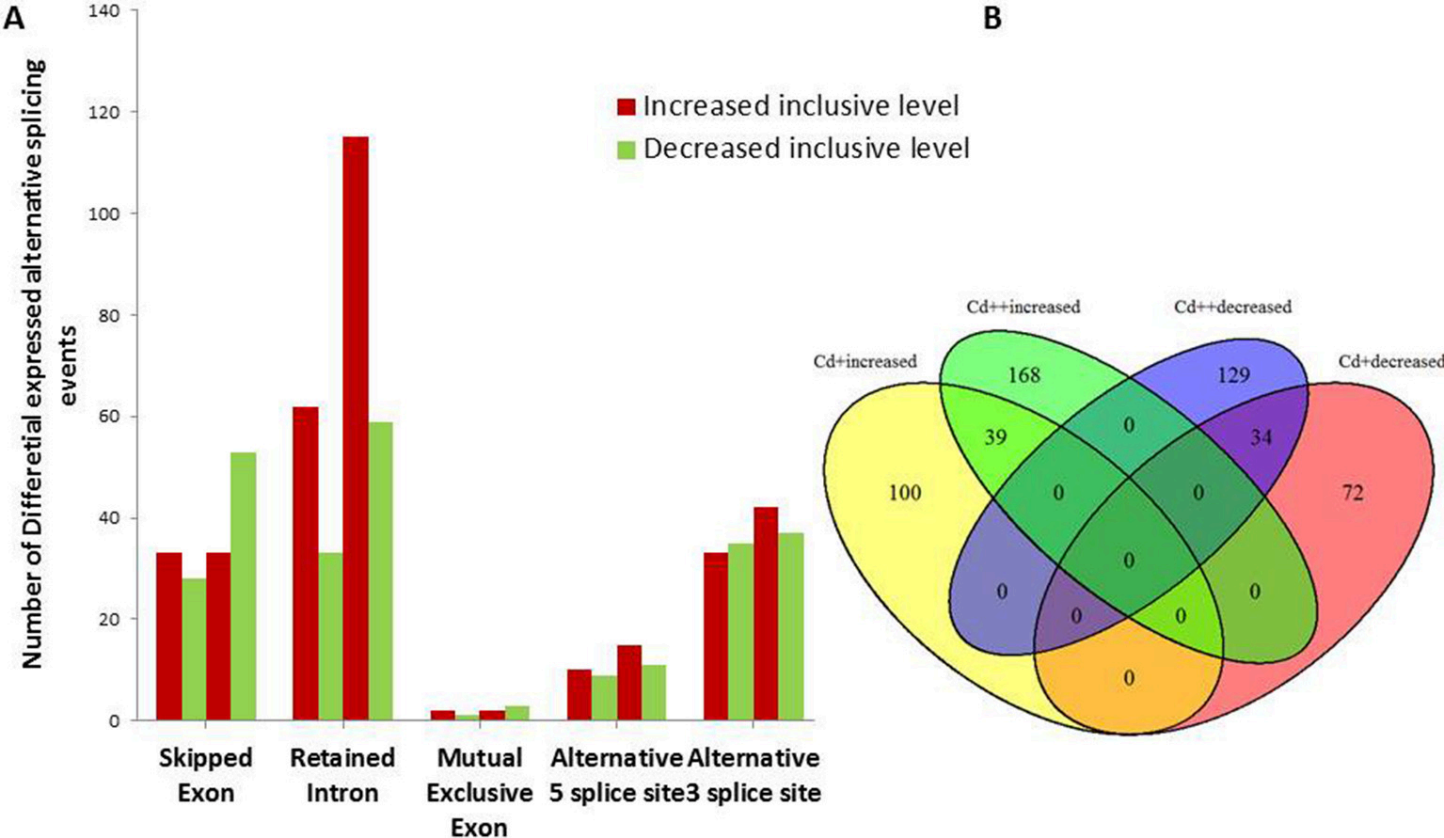
**Differential alternative splicing events. (A)** Summary of AS events with significant increased or decreased exon inclusive ratio between Cd-treated and control. **(B)** Venn diagram analysis showed shared 73 differentially expressed AS events in different conditions.

**Figure 9:**
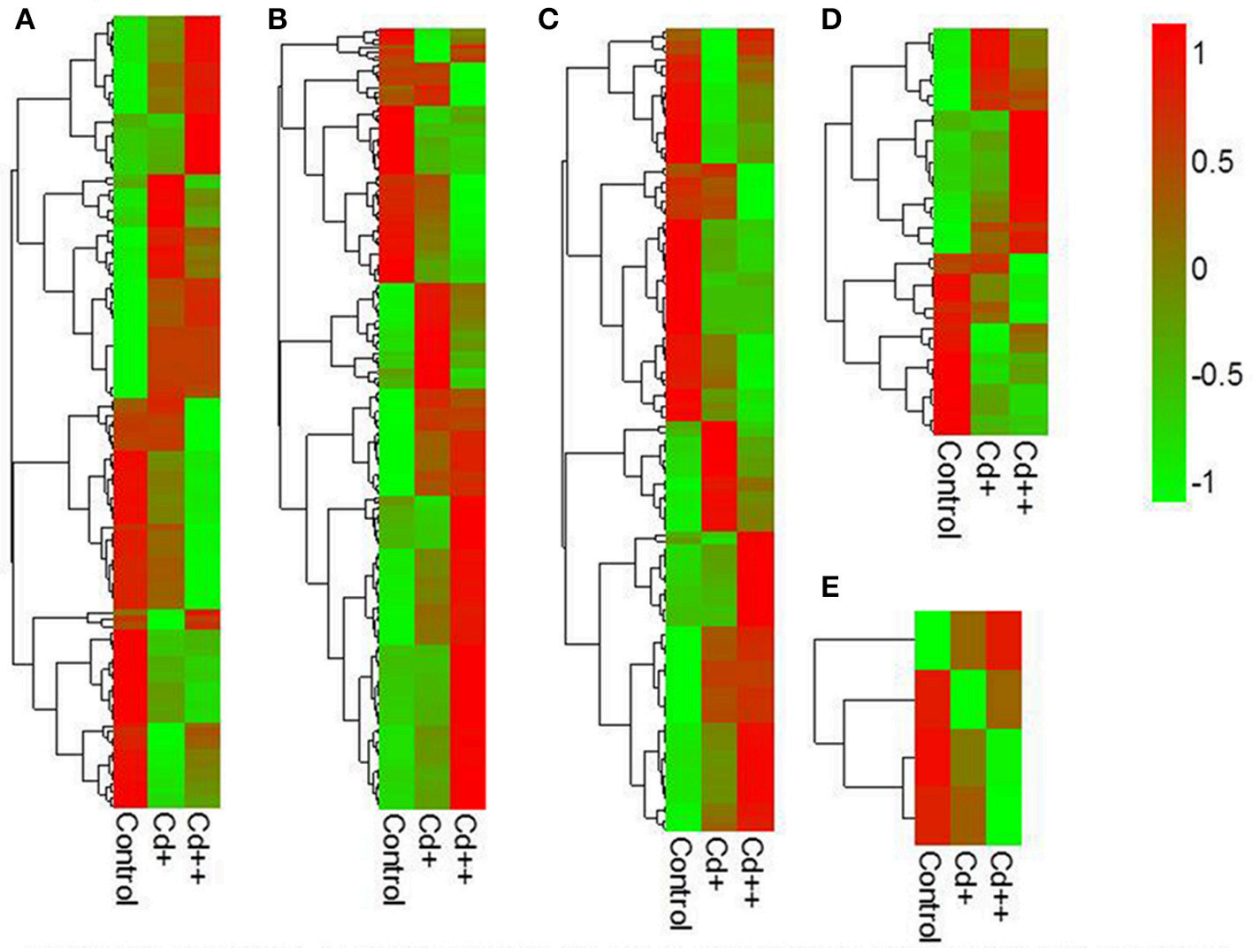
**Heatmaps of exon inclusive ratio between Cd-treated and control for differential alternative splicing events**. **(A)** Skipped exon. **(B)** Retained intron. **(C)** Alternative 3′ splice site. **(D)** Alternative 5′ slice site. **(E)** Mutually exclusive exon.

**Figure 10:**
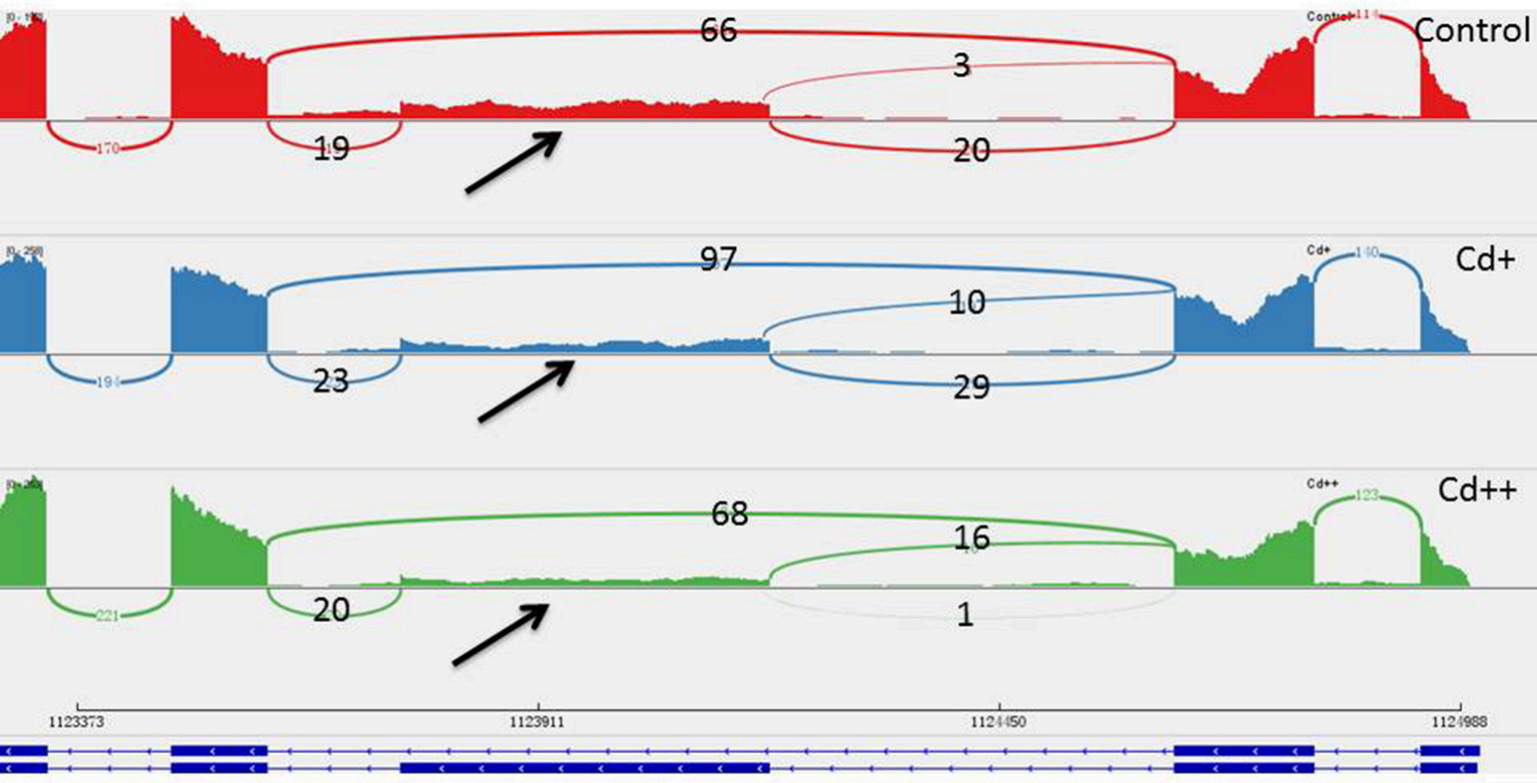
**One differential alternative skipped exon event of *Os04g0118900***. The arrow showed that the middle exon was differentially spliced between Cd-treated and control. Three numbers showed how many exon inclusive reads supported the upstream splice junction, the alternative exon itself, and the downstream splice junction and how many exon skipped reads supported the skipping splice junction that directly connects the upstream exon to the downstream exon.

**Figure 11:**
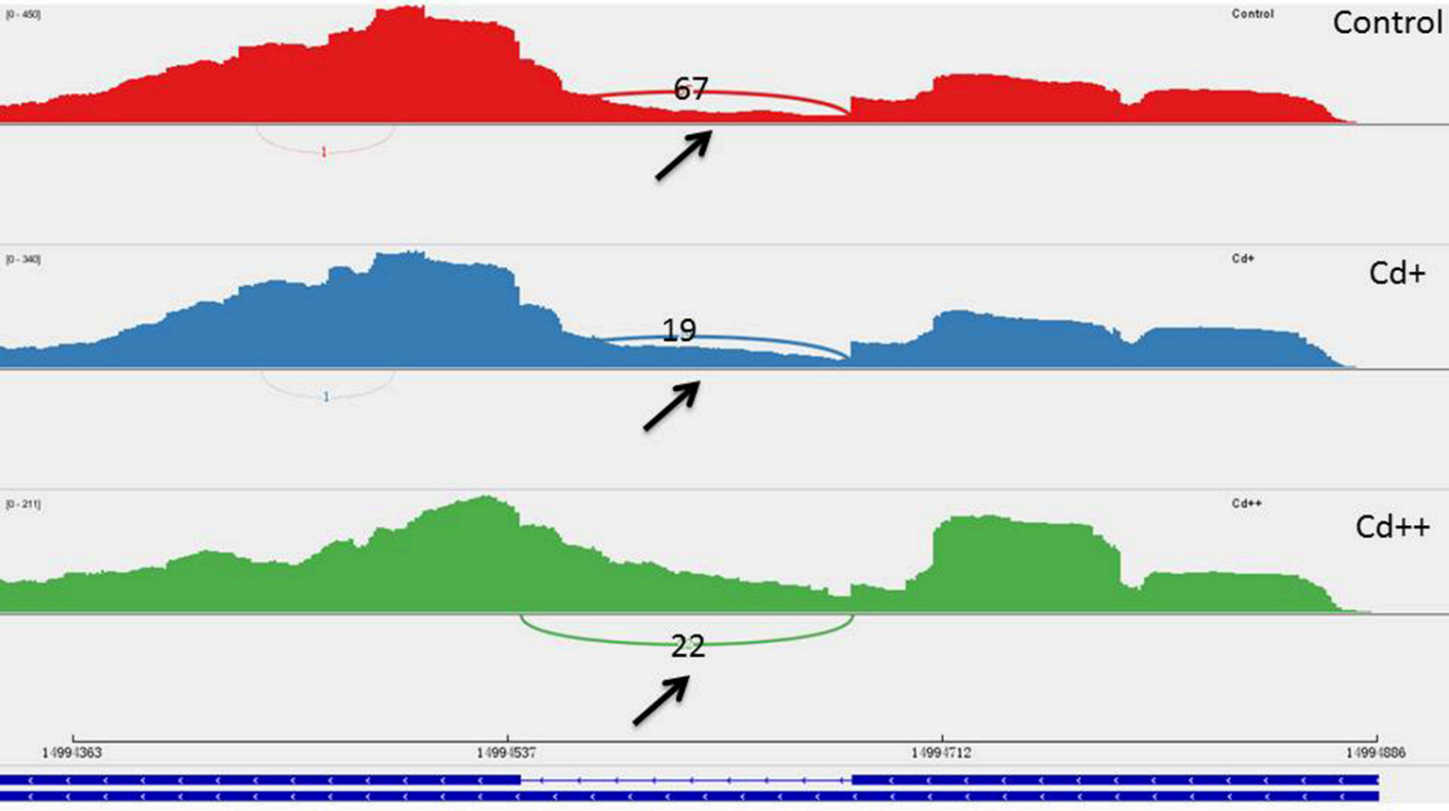
**One differential alternative retained intron event of *Os09g0417800***. The arrow showed that the intron was differential spliced between Cd-treated and control. Three number showed how many reads supported the middle intron between the upstream exon to the downstream exon was not retained.

To investigate the influence of Cd on alternative splicing regulation, expressed alternative splice events including skipped exon (SE), retained intron (RI), alternative 5′ splice site (A5SS), alternative 3′ splice site (A3SS), and mutual exclusive exons (MXE) in Control, Cd+ and Cd++ were identified using rMATS. A total of 8045 AS events were identified, of which 3488 AS events were commonly detected among three samples. RI, SE, and A3SS appear to be the most predominant AS events, relative to other AS event types, the most detected novel expressed SE events indicated that exon skipping greatly contributed to expressed transcript isoform diversity (Figures 7A–C). The 3488 AS events were distributed in 2415 genes. These AS genes were highly enriched in oxidation reduction, developmental process, response to hormone stimulus, signal transduction, and cell cycle (Supplemental Table S14). Using cutoff ≥ 0.1 for splicing differences and *P* ≤ 0.05, differential AS events were identified between Cd-treated and control samples (Figures 8, 9, Supplemental Tables S15–S24). We found 476 differential AS genes with 542 aberrant splicing events (Supplemental Table S25). Interestingly, only 8 genes were overlapped with DGEs (Supplemental Table S26). One example (Os04g0118900) corresponding to skipped exon and another (Os09g0417800) corresponding to retained intron with strong evidence of differential splicing based on their read coverage signal maps and exon-exon junction reads are shown in Figures 10, 11. Semi-quantitative RT-PCR validation performed in two selected transcripts confirmed their Cd-regulated AS pattern (Figure 3C). GO enrichment analysis indicated that 476 differential AS genes were highly enriched in oxidation reduction and cellular response to chemical stimulus (Supplemental Table S27). Two hundred and twenty-seven genes with differential RI were highly enriched in oxidation and signaling process (Supplemental Table S28). One hundred and twenty-one genes with differential SE had no significantly enriched biological processes (Supplemental Table S29).

## Discussion

Cd is one of the most toxic heavy metals; its toxicity in plants, animals and humans causes a major problem for crop production and food safety. Therefore, it is necessary to minimize the Cd concentration in edible portions of some crops, such as rice, one of the most important crops. Understanding Cd-responsive gene regulatory mechanisms can help us identify new ways to manipulate Cd accumulation and distribution in rice. High-throughput RNA sequencing and comprehensive transcriptomic analyses help us understand gene regulation and mechanisms for plant tolerance to heavy metals.

Although some studies have shown several genes involved in the response to Cd stress in rice (Ogawa et al., 2009; Zhang et al., 2012; Lin et al., 2013), a few RNA-Seq approaches have been used to investigate heavy metal stress at the whole transcriptome level. As an open system, RNA-Seq may provide an opportunity to identify novel lncRNAs and AS genes involved in the Cd stress response. Indeed, in this study, a large number of DEGs and differential AS genes was obtained, which will provide valuable information on transcriptional and post-transcriptional regulation of gene responses to Cd stress in rice.

### Common cadmium stress responses

Among the Cd DEGs, the overrepresented biological functional pathway genes are involved in cell wall formation, signaling transduction, ROS scavenging system etc. (Supplemental Tables S8–S11), that is consistent with previous studies (Mendoza-Cózatl et al., 2005; Ahsan et al., 2009; DalCorso et al., 2010; Lin and Aarts, 2012; Lin et al., 2013).

Cd can induce plant cell wall modifications, and the cell wall participated in preventing Cd transportation (Meyer et al., 2015; Wang et al., 2015a). Our data showed that four cell wall related-genes encoding sperm-coating glycoprotein (SCP)-like extracellular protein were up-regulated in rice roots during the Cd+ or Cd++ stress response. As a member of the pathogenesis-related (PR) protein family, SCP-like extracellular proteins are involved in cellular signaling processes with a putative Ca^2+^-chelating function (Fernández et al., 1997; Milne et al., 2003) and are induced during incompatible host-pathogen interactions or other stress conditions (Van Loon and Van Strien, 1999). They may play important functions in adaptation to biotic and abiotic stresses, including Cd stress. Four calmodulin encoding genes and two calmodulin-binding genes are up-regulated under Cd exposure suggesting they may directly involve in a complex network of signaling transduction that assists rice to effectively cope with Cd stress. In addition, one MAPKK gene (Os06g0191500) was detected in rice roots and was induced by Cd stress, suggest it may also be involve in signaling transduction pathway.

The ROS machine are usually activated by Cd stress, it is really the most overrepresented biological pathway present in the DEGs of our study (Supplemental Table S30). We identified 17 genes encoding GSTs, 2 genes encoding phenylalanine ammonialyase, 2 MT genes and 3 oxalate oxidase-like protein encoding genes were up-regulated in rice under Cd stress, whereas 4 Cysteine-rich polypeptide genes were down-regulated to avoid excessive consumption of cysteine, an ingredient of GSH (Supplemental Table S30). Separate enrichment analysis of GO term and KEGG pathway for up- and down-regulated genes indicated that peroxidase family genes had different expression patterns in response to Cd stress. Interestingly, 5 genes encoding class III peroxidase genes were significantly differential expressed, including 2 up-regulated (Os12g0111800, Os10g0107000) and 3 down-regulated (Os01g0294500, Os06g0490400, Os09g0323900). Similar phenomenon was also observed in one recent study, which showed that differential expression patterns of five candidate maize class III peroxidase genes under abiotic stress (Wang et al., 2015c). High Cd concentrations lead to an increase in the levels of oxygen-free radicals and glutathione peroxidase, which catalyzes the production of GSSG, thereby reducing the content of oxygen-free radicals. ROS scavenging enzymes or antioxidants, such as peroxidases and GST, can keep ROS levels under control and reduce their toxicity to plant growth.

Under stress conditions, some new proteins, such as heat shock proteins (Hsps), which are responsible for protein folding, assembly, translocation, and degradation in many cellular processes, can be induced for adaptation purposes. Hsps play a crucial role in plant tolerance to stress (Wang et al., 2004). Here, we detected 20 Hsp genes (Supplemental Table S30), most of which were up-regulated by Cd stress in rice roots. Similar results were found in rice roots of 10 and 25 μM Cd-treated and 5 μM Cu-treated samples (Ogawa et al., 2009; Lin et al., 2013), indicating that Hsps are strongly induced by heavy metal stress. Two Chitinase (Os06g0726200, Os10g0542900) was also induced, in addition to other damage-induced proteins reported previously.

### Transcriptional regulation of Cd

TFs play many roles in plant abiotic and biotic stress responses. TFs, such as WRKY, basic leucine zipper (bZIP), ethylene-responsive (ERF), and myeloblastosis protein (MYB), play a key role in controlling the expression of specific stress-related genes in response to Cd stress (DalCorso et al., 2010). WRKY, NAC, MYB, and AP2 genes were up-regulated in rice roots treated with 10 μM Cd for 3 h (Ogawa et al., 2009). In *Arabidopsis thaliana* roots, some genes encoding AP2 domain-containing protein, C2H2 zinc finger protein, HSF, and MYB TFs are specifically induced by Cd^2+^ but not Cu^2+^ (Weber et al., 2006). The expression analysis of six poplar C2H2 ZFPs showed their involvement in response to cold, salt, osmotic, and mechanical stresses (Gourcilleau et al., 2011). Wheat ZAT7 and ZAT12 belonging to C2H2 zinc finger protein (ZFP) family were shown to improve ROS tolerance in *Arabidopsis* and may improve Al-induced oxidative stress tolerance in wheat (Ali-Benali et al., 2013). An AP2/ERF transcription factor, *RAP2.6*, participates in ABA, salt and osmotic stress responses in *Arabidopsis* (Zhu et al., 2010). A stress-responsive NAC gene isolated from rice, *SNAC2*, can improve stress tolerance in rice (Hu et al., 2008). Wheat overexpressing a *TaNAC6* gene exhibit higher tolerance to stress (Xue et al., 2011). MYB genes can also respond to one or more stress treatments, such as high salt, PEG and exogenous ABA (Zhang et al., 2012). A number of WRKY genes are also activated by heavy metal lead stress in *Arabidopsis* (Liu et al., 2009). *WRKY38* from barley is involved in cold and drought stress responses (Marè et al., 2004). *TcWRKY53* from Cd-treated *Thlaspi caerulescens* is strongly induced by NaCl, drought, cold, and salicylic acid (SA) stresses (Wei et al., 2009). Five up-regulated WRKY genes including *OsWRKY71* (Os02g0181300), *OsWRKY28* (Os06g0649000), *OsWRKY22* (Os01g0820400), and *OsWRKY42* (Os02g0462800) were involved in plant defense response (Liu et al., 2007; Abbruscato et al., 2012; Chujo et al., 2013; Han et al., 2014). One up-regulated *OsWRKY72* (Os11g0490900) was involved in the abscisic acid signal and auxin transport pathway (Yu et al., 2010). These results indicate that TFs may be directly involved in abiotic stress responses. Currently, we are performing functional analyses of several TFs for their putative roles in heavy metal tolerance.

### Long non-coding RNAs

Long non-coding RNAs (lncRNAs) are a class of RNAs of more than 200 nt that do not encode proteins. The roles of lncRNAs in gene activation are poorly understood; some studies found that lncRNAs were key regulators in the cell and are involved in plant stress responses (Wilusz et al., 2009; Xin et al., 2011). In this study, the 9 lncRNA genes expressed showed good correlation with Cd concentration (Table 3). It has been determined that one type of lncRNA transcribed by RNA polymerase V in *Arabidopsis* can provide binding sites associated with other factors and regulate chromatin modification (Rowley et al., 2013). Using computational analysis and an experimental approach, 125 putative lncRNAs involved in the stress response in wheat and non-conserved in plants were identified (Xin et al., 2011). From these results, we can speculate that lncRNAs play an important role in plant resistance to various environment stresses. However, the biological functions of these lncRNAs have yet to be determined. Moreover, some of the lncRNAs are antisense to protein-coding genes in the 5′- or 3′-untranslated region (UTR); therefore, it is more likely that they function by regulating the expression of other genes, as well as processing into microRNAs to suppress downstream genes (Ariel et al., 2015). Therefore, the identification of Cd-regulated lncRNAs suggests that post-transcriptional regulation may also be involved in heavy metal stress responses.

### Alternative splicing

Alternative splicing (AS) is a vital regulatory mechanism used to increase transcriptome and proteome diversity and represents adaptation during evolution and stress (Staiger and Brown, 2013; Filichkin et al., 2015b). There are more examples of the positive effect and biological function of AS during stress. To our knowledge, there is limited information on the effects of heavy metal stress on AS regulation, especially in the case of global transcriptomic profiles induced by treatment with certain heavy metals. Our data presented here emphasize the importance of AS in the response to stress adaptation, including that to heavy metals. One recent study showed that Zn regulation of AS of one major facilitator superfamily (MFS) transporter is essential for Zn toxicity (Remy et al., 2014). Under excess Zn concentrations, intron retention of ZIF2 in the 5′-UTR resulted in a more stable transcript and led to increased production of proteins that function in Zn detoxification. This example is highly indicative of the involvement of functional AS in heavy metal stress responses. In the present study, three AS-regulated splicing factors under Cd stress (Os09g0491756, Os02g0146400, Os04g0118900; Supplemental Table S26), of which the typical arginine/serine-rich (SR) splicing factor gene (Os04g0118900) with significantly increased intron inclusive level indicated that several AS self-regulatory circuits of splicing regulatory factors play a unexpected role in in plant Cd stress response (Figure 10). It would be interesting to investigate the consequence on genome-wide gene expression using genetic material of the SR gene in future study. We also found that one WRKY transcription factor gene (Os09g0417800) was differential alternative spliced with significantly decreased exon inclusive level under different levels of Cd stress (Figure 11). We can speculate that alternative splicing of the WRKY transcription factor may lead to target regulation of downstream genes that is close associated with plant responses to Cd stress.

## Conclusions

Recent studies on the transcriptomes of plants under Cd stress using microarray analysis have identified Cd stress-responsible metabolic pathways, including ROS scavenging enzymes, heavy metal chelators and transporters. Our present study utilizing RNA-Seq will provide a more comprehensive analysis of transcriptome under Cd stress, and will show that, in addition to transcriptional regulation, post-transcriptional regulation may also play a vital role in plant adaptation to environmental Cd stress. Moreover, our findings will not only provide us a more complete picture of the regulatory network of the Cd stress response in plants, but will also provide us with the potential to produce Cd stress-tolerant crops.

## Author contributions

Manuscript draft: FH, QL, and LZ; Analyzing data: FH and LZ; Experiment: QL, LZ, and YC; Conception and supervision of the research: ZS and LZ.

### Conflict of interest statement

The authors declare that the research was conducted in the absence of any commercial or financial relationships that could be construed as a potential conflict of interest. The reviewer Yingchun Xu declares that, despite being affiliated with the same institute as the authors of the manuscript, the review process was handled objectively.

## Supplementary material

Supplemental Tables S1–S3 **Primers used for qRT-PCR analysis of Cd up- or down-regulated genes**. Primers used for Semi-quantitative PCR detection of alternative splicing event analysis. Primers used for qRT-PCR detection of lncRNAs expression.

Supplemental Tables S4–S5 **Up- and down-regulated DEGs by Cd+**.

Supplemental Tables S6–S7 **Up- and down-regulated DEGs by Cd++**.

Supplemental Tables S8–S11 **Gene Ontology and KEGG Pathway enrichment analysis results for commonly up-regulated or down-regulated DEGs between Cd+ and Cd++**. Gene Ontology and KEGG Pathway enrichment analysis results for Cd++ specific up-regulated or down-regulated DEGs.

Supplemental Table S12 **Transcription factors regulated by Cd**.

Supplemental Table S13 **Predicted lncRNAs using RNAseq data**.

Supplemental Table S14 **Gene Ontology and KEGG Pathway enrichment analysis results for detected AS genes**.

Supplemental Tables S15–S19 **Differential AS events between Cd+ and control, successively including skipped exon, retained intron, alternative 5′ splice site, alternative 3′ splice site and mutually exclusive exon**.

Supplemental Tables S20–S24 **Differential AS events between Cd++ and control, successively including skipped exon, retained intron, alternative 5′ splice site, alternative 3′ splice site and mutually exclusive exon**.

Supplemental Table S25 **Functional description for differential AS genes**.

Supplemental Table S26 **Gene Ontology enrichment analysis results for overlapped 8 genes between differential AS genes and DEGs**.

Supplemental Table S27 **Gene Ontology and KEGG Pathway enrichment analysis results for differential AS genes**.

Supplemental Table S28 **Gene Ontology enrichment analysis results for overlapped AS genes with differential RI**.

Supplemental Table S29 **Gene Ontology enrichment analysis results for overlapped AS genes with differential SE**.

Supplemental Table S30 **Cd stress responsive genes identified by previously reports, including Glutathione S-transferases, Peroxidase, PAL, Metalloendoproteinas, Oxalate oxidase, Cysteine-rich peptide, Terpene synthase, Terpenoid synthase, Heat shock protein, Chitinase, transporter**.

Supplemental Table S31 **qRT-PCR validation of two genes with no changes in expression under Cd+ stress**.
